# JCM-16021, a Chinese Herbal Formula, Attenuated Visceral Hyperalgesia in TNBS-Induced Postinflammatory Irritable Bowel Syndrome through Reducing Colonic EC Cell Hyperplasia and Serotonin Availability in Rats

**DOI:** 10.1155/2012/239638

**Published:** 2012-06-10

**Authors:** Hong-Yan Qin, Hai-Tao Xiao, Fung-Ping Leung, Zhi-Jun Yang, Justin C. Y. Wu, Joseph J. Y. Sung, Hong-Xi Xu, Xu-Dong Tong, Zhao-Xiang Bian

**Affiliations:** ^1^School of Chinese Medicine, Hong Kong Baptist University, Kowloon Tong, Hong Kong; ^2^Faculty of Medicine, The Chinese University of Hong Kong, Hong Kong; ^3^Faculty of Chinese Pharmacy, Shanghai University of Traditional Chinese Medicine, Shanghai 200032, China; ^4^China Academy of Chinese Medicine, Beijing, China

## Abstract

The present study aimed to investigate the analgesic effect of JCM-16021, a revised traditional Chinese herbal formula, on postinflammatory irritable bowel syndrome (PI-IBS) in rats. The trinitrobenzene sulfonic (TNBS) acid-induced PI-IBS model rats were orally administrated with different doses of JCM-16021 (1.2, 2.4, and 4.8 g/kg/d) for 14 consecutive days. The results showed that JCM-16021 treatment dose-dependently attenuated visceral hyperalgesia in PI-IBS rats. Further, the colonic enterochromaffin (EC) cell number, serotonin (5-HT) content, tryptophan hydroxylase expression, and mechanical-stimuli-induced 5-HT release were significantly ameliorated. Moreover, the decreased levels of mucosal cytokines in PI-IBS, especially the helper T-cell type 1- (T_h_1-) related cytokine TNF-*α*, were also elevated after JCM-16021 treatment. These data demonstrate that the analgesic effect of JCM-16021 on TNBS-induced PI-IBS rats may be medicated via reducing colonic EC cell hyperplasia and 5-HT availability.

## 1. Introduction

Irritable bowel syndrome (IBS), one of the most frequently reported functional disorders of the gastrointestinal tract, is characterized by chronic abdominal pain, and alterations in bowel habits. Clinically, there is a subset of IBS patients termed as postinfectious or PI-IBS; in these patients, IBS symptoms occur after an initial episode of acute gastrointestinal infection. The major clinical features of PI-IBS include loose stool with urgency, accelerated colonic transit, and decreased pain threshold, which are similar to those of diarrhea-predominant IBS (IBS-D) [1, 2]. Epidemiologic studies showed that IBS affects 5–10% of the populations, and IBS-D is reported as the most frequent subtype [3]. Prospective studies also showed that 3–36% of enteric infections may lead to the generation of new IBS symptoms. In recent years, more attention has been paid to PI-IBS because it is believed that the clear onset and well-defined pathophysiological changes will help us understand not only the PI-IBS but also other subtypes of IBS [4].

Visceral hypersensitivity is the leading hypothesis to explain painful symptoms suffered by IBS patients, although the mechanism is not fully understood [5–7]. Enterochromaffin (EC) cell hyperplasia and serotonin (5-HT) hyperactivity have been reported to play important roles in the development of visceral hypersensitivity in IBS patients [8–11], especially in IBS-D patients [12]. As an intercellular signaling molecule, 5-HT activates intrinsic and extrinsic primary afferent neurons, therefore, regulates the gastrointestinal sensitivity [13]. It has been reported that 5-HT excited nociceptive afferents and induced hypersensitivity in humans and rats through activating peripheral 5-HT_2A_ and 5-HT_3_ receptors [14–16]. Previous studies also showed that 5-HT potentiated visceral hypersensitivity by sensitizing certain peripheral transient receptor potential vanilloid (TRPV) function, such as TRPV1 and TRPV4, which are all critical integrators of various nociceptive stimuli [17–19]. Given the important role of 5-HT in developing and maintaining visceral hypersensitivity, 5-HT signalling pathways have been considered as a promising target for IBS treatment.

It is well known that about 95% of the total 5-HT is found in the gastrointestinal tract, with 90% in EC cells [20]. The rate-limiting enzyme of 5-HT synthesis, tryptophan hydroxylase (TPH), is found mainly located in EC cells [21]. In addition, EC cells have been regarded as the intestinal sensors that detected chemical, mechanical, or pathological stimuli in the lumen [22]. Following mucosal stimulation, 5-HT is quickly released from EC cells and activates serotonergic receptors located on nerve fibers. The actions of released 5-HT are terminated via being uptaken into the enterocytes by serotonin reuptake transporter (SERT) [23]. Because EC cells are the major cell type responsible for the synthesis, storage, and release of 5-HT in the gut, the number and function of EC cells play the important roles in intestinal homeostasis. Up to now, EC cell hyperplasia has been identified in a number of intestinal infections induced by bacteria, virus, and parasites [24–26], including PI-IBS patients [1, 27], but the underlying mechanisms remain unclear. Recently, studies on the linkage between EC cells and immune system showed that CD4^+^ T cells played an important role in the development of colonic EC cell hyperplasia in intestinal infection, and EC cells were influenced by helper T-cell subtype 1 (T_h_1) or subtype 2 (T_h_2) cytokine predominant environments [28, 29]. It has been proposed that any alterations in 5-HT availability, such as biosynthesis, release, or uptake, may contribute to the disordered gastrointestinal sensation and motility. Therefore, therapeutic strategies based on 5-HT system have been studied extensively [30, 31]. Nowadays, TPH inhibitor, 5-HT_3_ receptor antagonists, and 5-HT_4_ receptor agonists have been applied to treat functional gastrointestinal disorders [21, 32].

Though IBS seems not lead to more serious conditions in most patients, the multiple and persistent symptoms contribute to the high work absenteeism, high socioeconomic burden, and decline of life quality [33, 34]. Unfortunately, to date, there are not satisfactory treatments for IBS sufferers. The managements primarily aim at symptomatic relief [4, 35]. It is reported that only 14% of patients with IBS are completely satisfied with their current therapy [36, 37].

With the unsatisfactory treatment responses of western medicines, many IBS suffers turn to alternative treatment modalities, such as traditional Chinese medicine (TCM), acupuncture, qi-gong, and mind-body therapy [37, 38]. JCM-16021, a Chinese herbal formula composed of seven herbs, is modified based on a traditional formula (Tongxie Yaofang) for diarrhea and abdominal pain. In a randomized, double-blinded, and placebo-controlled trial, JCM-16021 has been proved to relieve symptoms in IBS patients [39]. Results from our previous studies also showed that JCM-16021 dose-dependently attenuated neonatal maternal separation-induced visceral hyperalgesia in rats through reducing colonic 5-HT synthesis and metabolism [40]. Whether JCM-16021 is beneficial for PI-IBS-related symptoms has not been investigated.

The present study hypothesized that JCM-16021 can attenuate visceral hyperalgesia in TNBS-induced PI-IBS rats, and the underlying mechanism may be mediated via reducing colonic EC cell hyperplasia and 5-HT availability. According to the results from our previous systematic review [41], the TNBS-induced PI-IBS rat model was used in this study. The PI-IBS rats were orally treated with JCM-16021 for 14 consecutive days, and then visceral sensation, EC cell number, 5-HT content, SERT, and TPH expression were investigated. In addition, mucosal cytokine productions were also investigated in order to identify whether there is an alteration in mucosal cytokine production of PI-IBS rats, and whether JCM-16021 has affect on it.

## 2. Materials and Methods

### 2.1. Materials

2,4,6-trinitrobenzenesulfonic acid (TNBS), parachlorophenylalanine (pCPA), sodium hyposulfite, and silver nitrate were purchased from Sigma-Aldrich (St. Louis, MO, USA). Chloral hydrate was purchased from Kou Hing Hong Scientific Supplies (Hong Kong, China). TNF-*α*, IFN-*γ*, IL-6, and IL-10 ELISA kits were purchased from eBioscience (San Diego, USA). The raw materials in JCM-16021 were all examined according to the quality control criteria of Chinese Pharmacopeia 2005 and controlled as previous report [40]. JCM-16021 granules were prepared as follows: raw materials at the total amount of tenfold of one day's dosage of JCM-16021 were mixed and macerated for 30 min, then decocted for 40 min for two times. The decocted solution was combined and precipitated in 65% ethanol for 16 h. The filtrates were finally dried in a vacuum at 70°C. The extraction efficiency of JCM-16021 granules was calculated according to the content of paeoniflorin, one of the active ingredients in JCM-16021.

### 2.2. Animals and Induction of PI-IBS Model

Sprague-Dawley male rats (aged 6 weeks with body weight around 220 g) were obtained from the Laboratory Animal Services Centre, The Chinese University of Hong Kong. Rats were maintained at 25°C under 12 h-12 h alternating light-dark cycle. Before colitis induction, rats were allowed to adapt the environment for 1 week. The PI-IBS rat model was developed according to previous report [42]. Briefly, after fasted for 24 hours, the rats were deeply anesthetized with chloral hydrate (350 mg/kg, i.p.). A plastic catheter (external diameter = 0.96 mm) was inserted into the descending colon at the depth of 8 cm from anus, then TNBS (5 mg/rat, 0.8 mL in 50% ethanol) saline solution was instilled slowly. The control group was treated with 0.8 mL saline instead of TNBS. After TNBS or saline administration, the rats were left on a mound of bedding in head-down position to prevent drug leakage. At 4 weeks after TNBS administration, visceral pain threshold pressure was measured. The rats with acquired visceral hyperalgesia (pain threshold pressure below 30 mmHg) were selected as the PI-IBS rats. All studies were carried out in accordance with the guidelines of the Committee on Use of Human and Animal Subjects in Teaching and Research, Hong Kong Baptist University.

### 2.3. Experimental Design

The first series of experiments aimed to test whether JCM-16021 can attenuate visceral hyperalgesia in TNBS-induced PI-IBS rats. Six groups of 12 rats were used. The PI-IBS rats were randomly divided into 5 groups and treated with water (Group 1), pCPA (TPH inhibitor, 150 mg/kg/d∗3 d, i.p., Group 2), and JCM-16021 (Group 3–5) at the dose of 1.2, 2.4, and 4.8 g/kg/d; and the normal control (Group 6) were treated with water. After 2 weeks treatment, 5 rats in each group were used to measure pain threshold pressure, and the rest of the rats were used for electromyography recording. In pCPA-treated group, pain threshold pressure was measured at 3 days after pCPA administration. Subsequently, the rats were sacrificed for sample collection. A 6 cm long proximal colon (1-2 cm from caecum) was harvested and divided into 3 parts, the proximal was fixed in 4% paraformaldehyde and embedded in paraffin; the middle part was collected for 5-HT content measurement and electron microscopic evaluation; the distal part was for western blotting analysis. The transverse colon mucosa was also collected for cytokine assay.

The second series of experiments aimed to evaluate the effects of JCM-16021 on EC cell hyperplasia, 5-HT synthesis, and 5-HT release in PI-IBS rats. Six groups of 5 rats were used in this experiment, and the group setting and treatments were as same as that of the first series of experiments. Differ from that of the first series of experiment, AWR test was not applied in order to obtain the baseline data that without colorectal distension (CRD) stimulus. The samples were collected and processed as same as that described in the first series of experiments.

### 2.4. Abdominal Withdrawal Reflex (AWR)

AWR test was performed as previously described [43]. Briefly, rats were lightly anesthetized with ether, then a 6 cm-long flexible latex balloon was inserted into the descending colon. The end of the balloon was secured at least 1 cm proximal to the anal verge. Rats were then allowed to recover for about 1 h, and colorectal distension was applied in increments of 5 mmHg until a visible contraction of the abdominal wall was observed by an investigator blinded to the treatment. The pain threshold pressure was defined as the intensity of colorectal distension that elicited an observable AWR, that is, a sudden and persistent abdominal muscle contraction with abdomen lift off the platform (Score 3). The pain threshold pressures of all groups were repeated five times with intervals of at least 5 minutes for recovery.

### 2.5. Electromyography (EMG) Recording

To evaluate the effects of JCM-16021 on visceral hyperalgesia in PI-IBS rats, visceral motor response to CRD was also applied in this study, and the experiment was conducted as previously described [44, 45]. Firstly, a surgical operation was made to implant the electrodes into the left external abdominal oblique muscles at 7 days before EMG recording. After the final drug administration, a flexible latex balloon was inserted into the rat descending colon, and the rats were allowed to accommodate for 1 h. Colorectal distension was initiated, and the EMG signal was amplified and filtered by Power Lab System. Graded colorectal distension (20, 40, 60, and 80 mmHg; 20 s duration; 2 min interstimulus interval) was carried out for total three cycles to each rat. The changes of area under the curve (AUC) were calculated using that during 20 s distension period over the 20 s baseline.

### 2.6. EC Cell Counting

Tissue sections (5 *μ*m thick) were deparaffinized and rehydrated for silver staining according to a method previously described [46]. Briefly, sections were incubated with 5% ammoniacal silver solution for 4 h at room temperature, then 2 h in 56°C and subsequently 12 h at room temperature in a dark humidified chamber. After rinsing with water, 5% sodium hyposulfite was added and incubated for 5 min at room temperature. The brown to black silver precipitates in the cytoplasm of EC cells were considered as a positive reaction. Five random fields at 200x magnifications were counted in each section by a researcher blinded to the treatment; the number of EC cells per mm^2^ of mucosa was quantified using Image J NIH software.

### 2.7. 5-HT Assessment

5-HT content in the colon tissue was assayed followed by our previously reported procedure [47]. Briefly, the colon segments were homogenized in 15% iced trichloroacetic acid for 2 min and stored at 4°C for 2 h. After centrifuge at 10,000 G for 15 min, the supernatant of each sample was filtered through 0.22 *μ*m filter and extracted with diethyl ether for five times. The prepared samples were added to derivatization solution (150 mM sodium tetraborate, 25% acetonitrile, 2 mM CFSE) and incubated for 30 min at room temperature in dark. The 5-HT content was analyzed by capillary electrophoresis with laser-induced fluorescence detection and expressed as nanogram per milligram (wet weight of the tissue).

### 2.8. Western Blot Analysis

The colonic tissues were homogenized and sonicated on ice for protein extraction. After determination of protein content, the samples were denatured at 100°C for 5 min. Proteins were separated by 10% SDS-PAGE and transferred to PVDF membrane (Bio-Rad, CA). After blocking with 5% nonfat milk, the membrane was incubated overnight at 4°C with rabbit anti-TPH antibody (1 : 1000, Santa Cruz, CA), then incubated with the appropriate secondary antibodies for 1 h at room temperature. The immunoreaction was detected using ECL Western blotting kit (ECL, Amersham, UK). Bands were visualized on Biomax X-ray film, and the optical density of each band was semiquantified by computer software (Image J.NIH). All detected protein bands were normalized to *β*-actin levels.

### 2.9. Immunofluorescence Staining for SERT

The tissue sections (6 *μ*m thick) were rinsed in PBS (pH. 7.4) and placed in polycarbonate staining jars (Kartell, Italy) filled with 10 mM sodium citrate buffer solution (pH. 6.0). After 15 min microwave exposure for antigen retrieval, nonspecific antibody binding was reduced by incubation of the tissues in a solution of 3% bovine serum albumin, 0.5% Triton X-100 in PBS. Sections were incubated with rabbit anti-SERT polyclonal antibody (1 : 500, Calbiochem, CA) in PBS containing 0.5% Triton X-100 overnight at 4°C. After rinsed in PBS, the sections were incubated with Alexa fluor 488-conjugated secondary antibody (1 : 200, Invitrogen) for 2 h at room temperature, and then rinsed and coverslipped. Sections were observed with a laser scanning microscope (Olympus Fluoview FV1000), captured with 400x, and analyzed using Image J NIH software. The optical densities in colonic mucosa were measured after background subtracting. The total SERT immunofluorescence densities from 5 random fields per section were calculated.

### 2.10. Electron Microscopic Evaluation

The colon segments were fixed for 24 h at 4°C in the fixative containing 4% paraformaldehyde and 0.2% picric acid in 0.1 M PB then washed and embedded in gelatin. Thin sections were cut and picked up on the 200-mesh grids. The sections were stained with uranyl acetate and lead citrate. Examination and photography of sections were carried out using Philips CM 10 transmission electron microscope (Philips Scientifics, Netherlands). At the ultra-structural level, EC cells were distinguished from other enteroendocrine cells by the pleomorphic nature of their secretory granules [48]. The EC cell in which there were many clear secretory granules without cores or granules with eccentric cores was considered as the activated one with excessive 5-HT release [49].

### 2.11. ELISA Assay

The mucosal samples were lysed with Tris EDTA (10 mM Tris-HCl, 1 mM EDTA, pH 7.4) containing 0.5% Triton X-100 and protease inhibitors, and then the samples were homogenized and sonicated on ice. After centrifuge at 10,000 G for 10 min at 4°C, the supernatant was collected and stored at −80°C until use. Mucosal cytokine levels of TNF-*α*, IFN-*γ*, IL-6, and IL-10 were determined using ELISA kits according to the manufacturer's protocol. The results were expressed at pg/mg total protein.

### 2.12. Statistical Analysis

Data are presented as mean ± S.E.M. Statistical analysis was conducted using SPSS 12.0 Software. Differences between two groups were analyzed with Student *t*-test. When multiple groups were compared, data were analyzed using one-way ANOVA followed by Student-Newman-Keuls (SNK) test. Differences were considered significant when *P* < 0.05.

## 3. Results

### 3.1. Analgesic Effect of JCM-16021 in PI-IBS Rats

**Figure 1:**
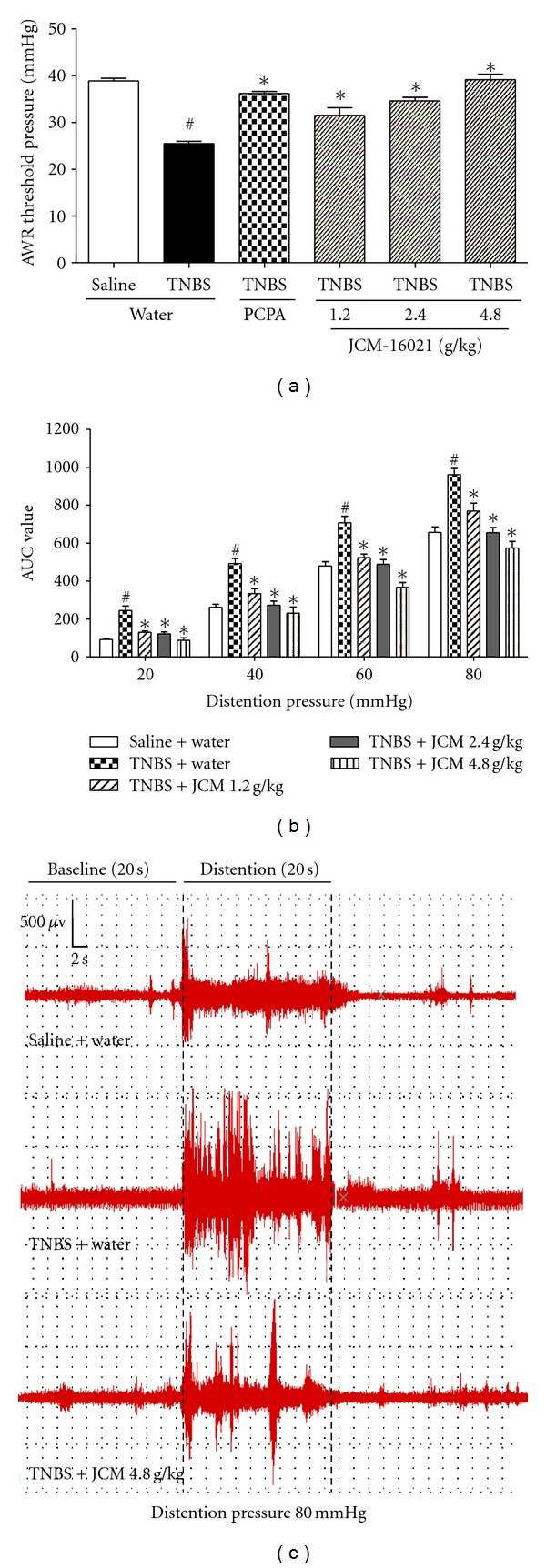
Effects of JCM-16021 on visceral hypersensitivity of PI-IBS rats. (a) depicts the analgesic effect of JCM-16021 in PI-IBS rats in terms of pain threshold pressure, while (b) depicts the effect of JCM-16021 on visceral motor response to graded CRD in PI-IBS rats. The representative EMG graphs from normal rats, PI-IBS rats, and high-dose JCM-16021 treated rats in a 20 s noxious (80 mmHg) colorectal distension period are shown in (c). Data are presented as mean ± S.E.M., *n* = 5 per group in AWR test, *n* = 8 per group in EMG recording test. ^#^ *P* < 0.05 versus normal rats, *P < 0.05, versus PI-IBS rats (*t*-test).

As shown in Figure 1(a), the pain threshold pressures in PI-IBS rats (TNBS + water treated rats) were significantly decreased when compared to that of the control (saline + water treated rats, *P* < 0.05). After pCPA treatment, the pain threshold pressures in PI-IBS rats were significantly elevated (*P* < 0.05), suggesting that 5-HT played an important role in the development of visceral hypersensitivity. After JCM-16021 treatment, the pain threshold pressures were significantly and dose-dependently elevated when compared to that of the PI-IBS rats (*P* < 0.05), indicating that JCM-16021 has analgesic effect in PI-IBS rats. Consistent with the findings from AWR test, the results from EMG recording (Figures 1(b) and 1(c)) also showed that visceral motor responses to graded CRD in PI-IBS rats were significantly increased when compared to that of the control (*P* < 0.05). After JCM-16021 treatment, the visceral motor responses to graded CRD in PI-IBS rats were decreased significantly in a dose-dependent manner (*P* < 0.05).

### 3.2. Effects of JCM-16021 on Colonic EC Cell Number and 5-HT Content in PI-IBS Rats

**Figure 2:**
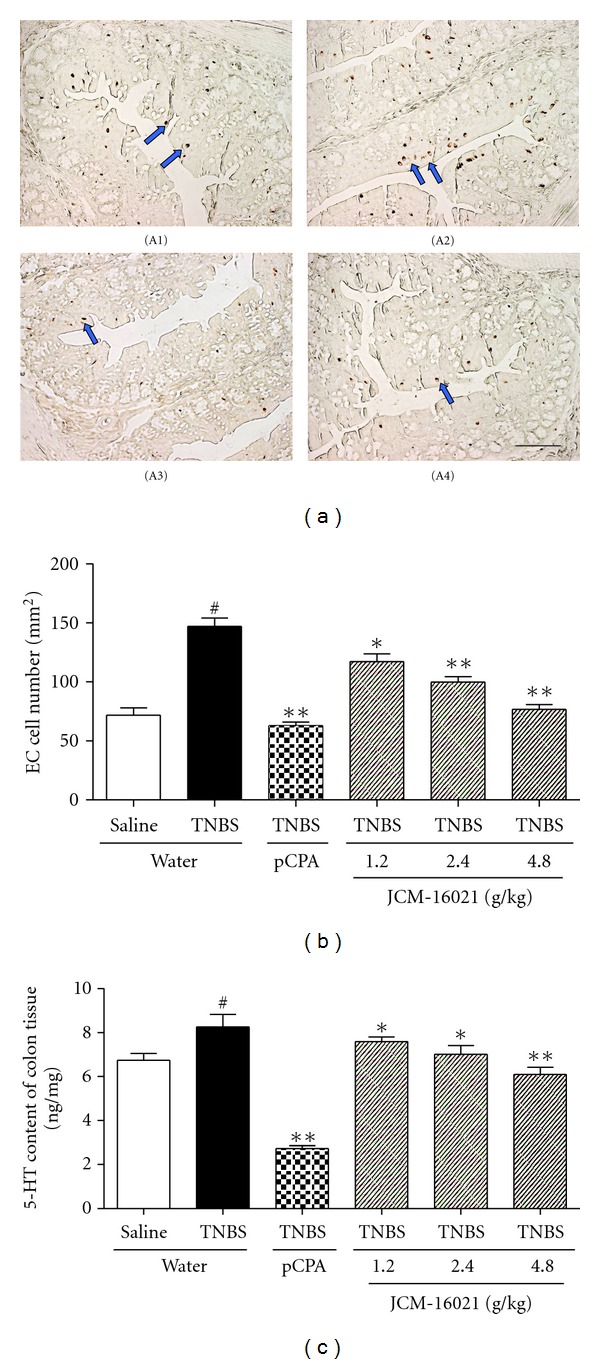
Effects of JCM-16021 on enterochromaffin (EC) cell density and 5-HT content in the colon tissue of PI-IBS rats. (a) shows the representative EC cells (arrowhead) in colonic mucosa of the (A1) normal rats, (A2) PI-IBS rats, (A3) pCPA treated rats, and (A4) high dose JCM-16021 treated rats (Scale bar, 200 *μ*m). Statistical graph of EC cell density is shown in (b), and 5-HT content in (c). Data are presented as mean ± S.E.M., *n* = 5 per group. ^#^ *P* < 0.05 versus normal rats, *P < 0.05, **P < 0.01 versus PI-IBS rats (*t*-test).

As shown in Figure 2, the colonic EC cell density and 5-HT content were both significantly increased in PI-IBS rats (~51% in EC cell density, ~23% in 5-HT content) when compared to that of the control (*P* < 0.05), suggesting the occurrence of EC cell hyperplasia in PI-IBS rats. Compared with the PI-IBS rats, JCM-16021 treatment significantly and dose-dependently decreased the colonic EC cell density (20%~48%) and 5-HT content (8%~26%) in PI-IBS rats, suggesting that JCM-16021 can reduce colonic EC cells hyperplasia of PI-IBS rats. PCPA treatment also significantly decreased the colonic EC cell density and 5-HT content in PI-IBS rats (~57% in EC cell density, ~62.5% in 5-HT content, *P* < 0.05).

### 3.3. Effect of JCM-16021 on Colonic TPH Expression in PI-IBS Rats

**Figure 3:**
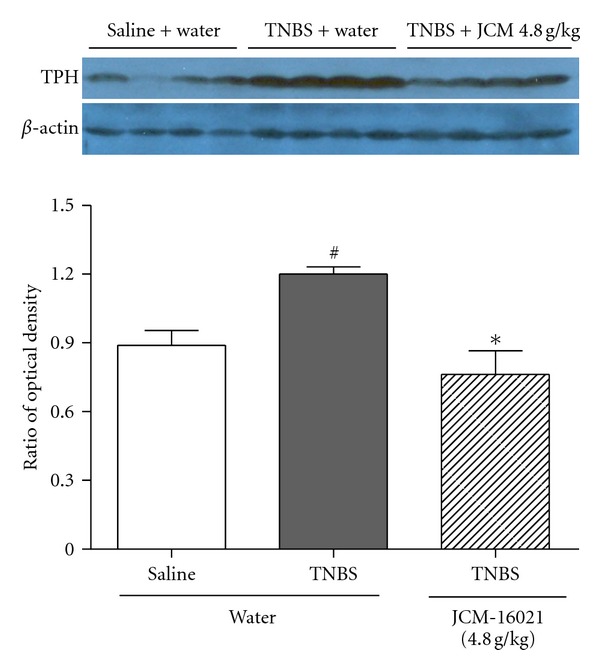
Effect of JCM-16021 on colonic TPH expression in PI-IBS rats. Western immunoblots of TPH and the statistical analysis of protein level showed that JCM-16021 significantly decreased the colonic TPH expression in PI-IBS rats. Data are shown as mean ± S.E.M., *n* = 4 per group. ^#^ *P* < 0.05 versus normal rats, *P < 0.05 versus PI-IBS rats (*t*-test).

Given the important role of the rate-limiting enzyme in 5-HT synthesis, colonic TPH expression was further evaluated by Western blot technique. As shown in Figure 3, TPH protein expression in PI-IBS rats was significantly increased (~26%) when compared to that of the control (*P* < 0.05), while high-dose JCM-16021 treatment significantly reduced the TPH expression in PI-IBS rats (~36%, *P* < 0.05).

### 3.4. Effect of JCM-16021 on Mucosal SERT Expression in PI-IBS Rats

**Figure 4:**
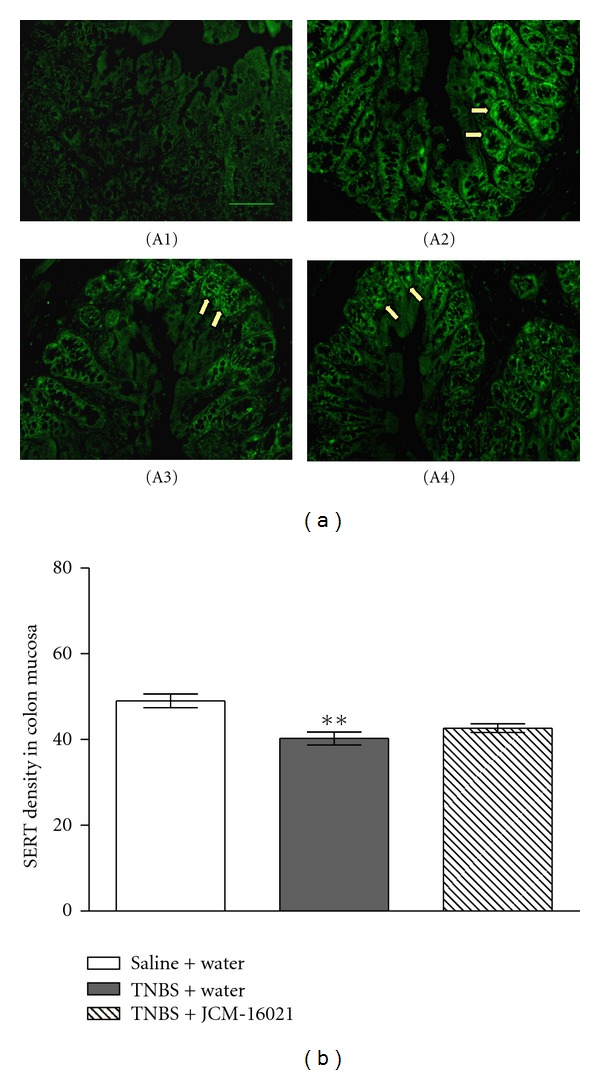
Effect of JCM-16021 on mucosal SERT expression in PI-IBS rats. (a) shows the immunofluorescence micrographs of (A1) negative control (primary antibody omitted) and the positive SERT expressions (arrowhead) in the mucosa of (A2) normal rats, (A3) PI-IBS rats, and (A4) high dose JCM-16021 treated rats (Scale bar, 200 *μ*m). Statistical analysis of intensity of SERT immunoreactivity is shown in (b). Data are shown as mean ± S.E.M., *n* = 5 per group. **P < 0.01 versus normal rats (*t*-test).

As shown in Figure 4, the intensity of SERT immunoreactivity in colonic mucosa of PI-IBS rats was significantly decreased when compared to that of the control (40.3 ± 3.1 versus 49.0 ± 3.2; *P* < 0.05), suggesting that TNBS-induced PI-IBS rats also has the decreased SERT expression in the colon. After treatment of JCM-16021, there were no significant differences in the expression of SERT immunoreactive intensity between PI-IBS rats and JCM-16021-treated rats (40.3 ± 3.1 versus 42.6 ± 2.1; *P* < 0.05), suggesting that JCM-16021 treatment has little effect on the decreased SERT expression in PI-IBS rats.

### 3.5. Effect of JCM-16021 on Mechanical Stimuli-Induced 5-HT Release in PI-IBS Rats

**Figure 5:**
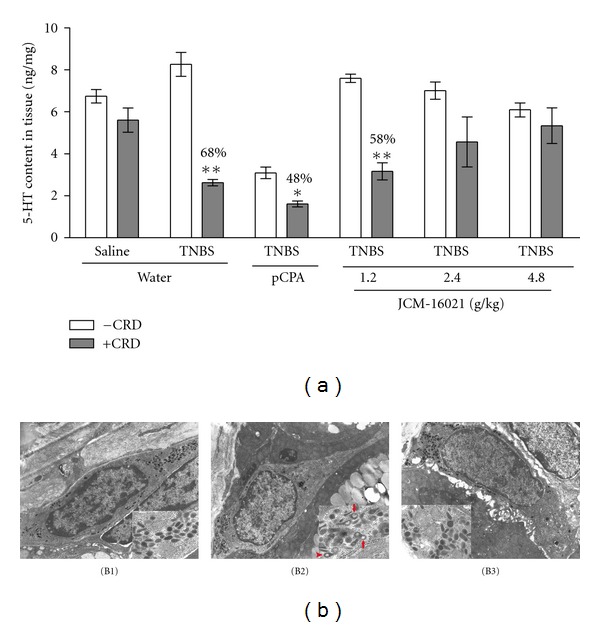
Effect of JCM-16021 on mechanical stimuli (CRD) induced 5-HT release in PI-IBS rats. (a) shows the statistical analysis of colonic 5-HT content in PI-IBS rats with or without CRD treatment. (b) shows the representative electron microsgraphs of EC cells and their secretory granules (inset) from the (B1) normal rats, (B2) PI-IBS rats, and (B3) high dose JCM-16021 treated rats (×8900; inset: ×10000). Activated EC cell is characterized by the clear secretory granules without cores (red arrow), or secretory granules with eccentric cores (red arrowhead) in it. Data are shown as mean ± S.E.M., *n* = 5 per group. *P < 0.05, **P < 0.01 versus the same group without CRD treatment (*t*-test).

To identify whether mechanical stimuli (CRD) can induce excessive 5-HT release in PI-IBS rats and the effect of JCM-16021 on it, colonic 5-HT content was evaluated in rats with or without AWR test. As shown in Figure 5(a), after CRD application, the 5-HT content of normal rats reduced by ~17% when compared to that without CRD, but no statistical differences were found between these two groups; while in PI-IBS rats, 5-HT content dramatically decreased by ~68% after CRD application (*P* < 0.01). In pCPA-treated rats, CRD also induced marked decrease of 5-HT content in PI-IBS rats (~48%, *P* < 0.05), even though the baseline 5-HT content has been lowered below the normal level. After high and medium dose of JCM-16021 treatment, CRD did not induce significant decreases of 5-HT content (~35% and ~12%) when compared to that without CRD application, suggesting that the treatment of high and medium dose of JCM-16021 may attenuate CRD-induced excessive 5-HT release in PI-IBS rats. Results from electronic microsgraphs also showed that the secretory granules within EC cells of PI-IBS rats were characterized by clear secretory granules without cores or empty vesicles after CRD application, indicating that there may be an excessive 5-HT release in PI-IBS rats after mechanical stimulation. In high-dose JCM-16021-treated group, the secretory granules within EC cells showed little empty vesicles when compared to that of the PI-IBS rats (Figure 5(b)).

### 3.6. Effect of JCM-16021 on Mucosal Cytokine Production in PI-IBS Rats

**Figure 6:**
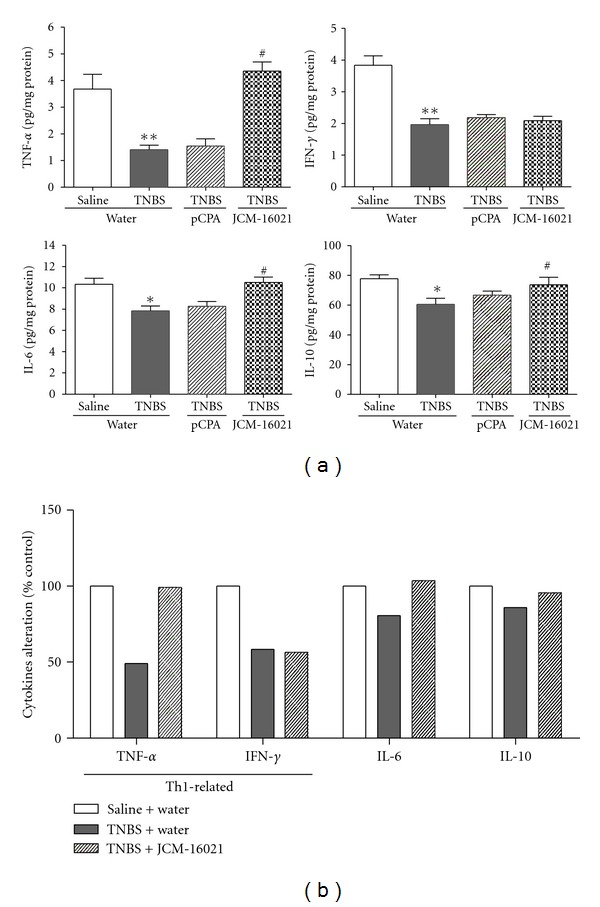
Effect of JCM-16021 on mucosal cytokines production in PI-IBS rats. Statistical graphs about mucosal cytokines production are shown in (a). The rates of cytokines alteration are shown in (b). Data are shown as mean ± S.E.M., *n* = 5 per group. *P < 0.05, **P < 0.01 versus normal rats, ^#^ *P* < 0.05 versus PI-IBS rats (*t*-test).

As shown in Figure 6(a), the levels of TNF-*α*, IFN-*γ*, IL-6, and IL-10 in the colonic mucosa of PI-IBS rats were all significantly decreased when compared to that of the normal rats (*P* < 0.05). High dose of JCM-16021 treatment significantly elevated the levels of TNF-*α*, IL-6, and IL-10, but not IFN-*γ*, in PI-IBS rats, suggesting that JCM-16021 may modulate mucosal cytokines production in PI-IBS rats. In pCPA-treated rats, the cytokine levels were not changed when compared to that of the PI-IBS rats, indicating that 5-HT depletion may have little effect on cytokines production of PI-IBS rats. As shown in Figure 6(b), even though the detected cytokines were all decreased significantly in PI-IBS rats, the levels of Th1-related cytokines, TNF-*α* and IFN-*γ*, were decreased more obviously (51% decrease in TNF-*α*, 42% decrease in IFN-*γ*) than others (14% decrease in IL-10, 19% decrease in IL-6).

## 4. Discussion

This study reveals that Chinese herbal formula JCM-16021 can dose-dependently attenuate visceral hyperalgesia in TNBS-induced PI-IBS rats, and this effect is mediated through reducing colonic EC cell hyperplasia and 5-HT availability. Moreover, our results also demonstrate that JCM-16021 treatment can upregulate the decreased levels of certain mucosal cytokines, especially the T_h_1-related cytokines, in PI-IBS, which may contribute to the therapeutic effect of JCM-16021.

As a neurotransmitter, 5-HT plays an important role in the perception and processing of nociceptive stimuli in the gastrointestinal tract. It is well known that EC cell hyperplasia and 5-HT hyperactivity are involved in the development of visceral hypersensitivity [8–11]. Results from this study showed that JCM-16021 treatment significantly and dose-dependently attenuated visceral hyperalgesia in PI-IBS rats, and this effect was concomitant with the decreased colonic EC cell number and 5-HT availability. Treatment of pCPA, the inhibitor of rate-limiting enzyme in 5-HT synthesis, dramatically decreased colonic EC cell number and 5-HT content in PI-IBS rats; and this effect was consistent with the attenuated visceral hyperalgesia in PI-IBS rats. All these results indicate that the decreased EC cell number and 5-HT content induced by JCM-16021 treatment may be responsible for the attenuated visceral hyperalgesia in PI-IBS rats.

In this study, pCPA treatment resulted in dramatic decrease of 5-HT content in PI-IBS rats. It is notable that 5-HT content in pCPA-treated rats was depleted seriously, but no differences were found in visceral pain threshold pressure between pCPA-treated rats and the normal rats. We speculated that the remained 5-HT content, even below the normal level, may be enough to fulfill the sensory reflex [50]. Moreover, as proposed by P. P. Bertrand and R. L. Bertrand [51], the adaptive changes of gastrointestinal tract to the environment and the overlapping functions of numerous gastrointestinal hormones may explain why 5-HT depletion in pCPA-treated rats did not cause marked change in pain sensation. Interestingly, we also found that the EC cell number in PI-IBS rats was significantly decreased after pCPA treatment. Current results cannot explain whether such results are related with the reduced EC cell markers or the total EC cell number. Further researches are needed.

It is well known that the main source of tissue 5-HT content is consisted of synthesized 5-HT in EC cells and the released 5-HT yet has not been taken up by SERT [9]. In this study, we further investigated the effect of JCM-16021 on TPH and SERT expression. The results indicated that colonic TPH expression was increased in PI-IBS rats; and high dose of JCM-16021 treatment significantly reduced the TPH expression in PI-IBS rats, which were consistent well with the alterations of EC cell number and 5-HT content. Moreover, the expression of mucosal SERT was found significantly decreased in PI-IBS rats, and this result was in agreement with the previous findings which showed the decreased SERT expression in IBS patients [30] and IBS animal models [52]. High dose of JCM-16021 treatment did not show any effect on the decreased SERT expression in PI-IBS rats, indicating that JCM-16021 treatment can decrease colonic EC cell number, 5-HT content, and TPH expression, but not SERT expression, in PI-IBS rats.

Knowing that the excessive availability of 5-HT are mainly come from increases of EC cell number, reduction of SERT expression, or increases of stimuli-induced 5-HT release [32], we further investigated the mechanical stimuli CRD-induced 5-HT release in PI-IBS rats. Our results showed that colonic 5-HT content and secretory granules within EC cells were dramatically reduced in PI-IBS rats after CRD application, suggesting that there is an excessive 5-HT release under mechanical stimulation in PI-IBS rats. Results from previous studies have shown that the postprandial 5-HT content in IBS-D and PI-IBS patients were significantly increased [8, 53], and 5-HT metabolites/5-HT ratios in PI-IBS patients were decreased [54]. These results provide the evidences that there is an excessive 5-HT release in IBS patients. As shown in this study, JCM-16021 treatment dose-dependently attenuated CRD-induced reduction of 5-HT content, and the secretary granules within EC cells were also increased in high-dose JCM-16021-treated rats. These results indicate that JCM-16021 administration can attenuate the mechanical stimuli-induced excessive 5-HT release in PI-IBS rats.

To date, the underlying mechanisms of EC cell hyperplasia in PI-IBS are unknown, but they are considered have close correlation with CD4^+^ T lymphocytes, especially the T_h_1/T_h_2 balance [29, 32]. Previous studies have shown that EC cell number and 5-HT content to the same infectious agent were influenced by T_h_1 or T_h_2 cytokine predominance, and the increased colonic EC cells and 5-HT content was found in deficient mice with impaired T_h_1 cytokine production [29]. Complemented with these findings, our results showed that TNBS-induced PI-IBS rats had colonic EC cell hyperplasia, and there is a dramatically reduced production of T_h_1-related cytokines in the colonic mucosa of PI-IBS rats. This study did not provide explanation why the levels of mucosa cytokines in PI-IBS rats were all decreased, but this result is consistent well with the findings from clinical studies, which showed lowered levels of intestinal cytokines in IBS patients [55]. May the lowered levels of mucosal cytokines of IBS patients result from the suppressed immune response or untriggered immune response after inflammation/infection? Is there an imbalanced T_h_1/T_h_2-related cytokines production in the colonic mucosa of PI-IBS rats? More studies are needed to explore these questions. In the present study, we found that JCM-16021 treatment significantly elevated the levels of reduced cytokines, especially the T_h_1-related cytokines, suggesting that JCM-16021 may have the potential effect on regulating immune system, which may be responsible for the reduced EC cell hyperplasia in PI-IBS rats. It is well known that most TCM remedies are formulated by using individual herbs in combination. Under the guidance of traditional theory, the different herbs of certain formula are thought to increase therapeutic efficacy and reduce adverse effects simultaneously through multiple targets and biological pathways [56]. To identify the main active constituents of JCM-16021 that are responsible for the therapeutic effects observed in this study, further work is need.

In conclusion, the analgesic effect of JCM-16021 on TNBS-induced PI-IBS rats may be mediated via reducing colonic EC cell hyperplasia and 5-HT availability, and the elevated production of mucosal T_h_1-related cytokines TNF-*α* may be responsible for the alterations induced by JCM-16021 in PI-IBS rats. The results from the present study give further evidences for the analgesic effect of JCM-16021 in IBS rats also provide preliminary finding for the reduced production of T_h_1-related cytokines in the colonic mucosa of PI-IBS rats.
